# Radiomics at a Glance: A Few Lessons Learned from Learning Approaches

**DOI:** 10.3390/cancers12092453

**Published:** 2020-08-29

**Authors:** Enrico Capobianco, Jun Deng

**Affiliations:** 1 Institute for Data Science and Computing, University of Miami, Coral Gables, FL 33146, USA; 2 Department of Therapeutic Radiology, Yale University School of Medicine, 15 York Street, New Haven, CT 06510-322, USA; jun.deng@yale.edu

**Keywords:** radiomics, machine learning, integrative inference approaches, predictive modeling

## Abstract

**Simple Summary:**

Radiomics has become a prominent component of medical imaging research and many studies show its specific value as a support tool for clinical decision-making processes. Radiomic data are typically analyzed with statistical and machine learning methods, which change depending on the disease context and the imaging modality. We found a certain bias in the literature towards the use of such methods and believe that this limitation may influence the capacity of producing accurate and reliable decisions. Therefore, in view of the relevance of various types of learning methods, we report their significance and discuss their unrevealed potential.

**Abstract:**

Processing and modeling medical images have traditionally represented complex tasks requiring multidisciplinary collaboration. The advent of radiomics has assigned a central role to quantitative data analytics targeting medical image features algorithmically extracted from large volumes of images. Apart from the ultimate goal of supporting diagnostic, prognostic, and therapeutic decisions, radiomics is computationally attractive due to specific strengths: scalability, efficiency, and precision. Optimization is achieved by highly sophisticated statistical and machine learning algorithms, but it is especially deep learning that stands out as the leading inference approach. Various types of hybrid learning can be considered when building complex integrative approaches aimed to deliver gains in accuracy for both classification and prediction tasks. This perspective reviews some selected learning methods by focusing on both their significance for radiomics and their unveiled potential.

## 1. Introduction

### 1.1. Radiomics

Driven by the recent advancement of precision medicine, both pathology and radiology have undergone substantial transformation. Among the most noticeable factors inducing change, there is the centrality assigned to data-driven integrative modeling approaches specifically designed to leverage quantitative imaging. These aspects have characterized the field of radiomics, a discipline strongly based on developing methods and algorithms able to reveal subtle disease marks by processing features extracted from medical images.

A recent review presenting the current research hotspots in radiomics [1] showed a concentration of applications in certain diseases areas and a prevalence of positive versus negative results, together with another two emerging aspects: (a) The dominant presence of non-clinical researchers and (b) the preferential choice of traditional statistical techniques (LASSO, logistic regression) in dealing with feature selection. This latter point is quite surprising and suggests that the spectrum of radiomic approaches (from handcrafted to machine learning (ML) driven) may require further consideration.

Radiomics uses a variety of ML methods that support inference and that may work standalone or be cast within integrative approaches, depending on the complexity of the context under study (cancer, diabetes, etc.). The progresses that have been made are mostly referred to: (i) Extracting computerized features from radiologic imaging, (ii) associating image features with molecular phenotypes (radiogenomics), and (iii) determining the relevance of radiologic features associated with pathologic phenotypes (radiopathomics).

In writing this perspective, our goal is twofold: To illustrate the relevance/significance of various types of learning methods in some of the current directions covered by radiomic studies and to discuss the potential that has not yet been revealed.

### 1.2. Directions in Radiomics

#### 1.2.1. Pathology

There are several challenges in the analysis of histopathological digital images, starting from the presence of biases, in relation to data quality, sampling strategy, and class labeling (see, for instance, [2]), and continuing with the specific analysis (pixel-wise, patch-level, voxel-wise, etc. [3,4]) that is run to detect and/or remove the biases and thus improve the generalization power. The advent of ML and deep learning (DL) in the digital pathology field has complemented the activity of existing statistical feature-driven methods, also simplifying massive image data classification tasks. Usual assessments include computing prediction probabilities that explain the patch-level model. For deep classification purposes, small-sized images (256 × 256 pixels) are often used as input such that large-sized images are resized into smaller ones. Clearly enough, increasing the input image size corresponds to (a) increasing the parameters to be estimated and (b) augmenting both computational power and memory burden. However, resizing may lead to side effects, such as loss of information at the cellular level and reduced identification accuracy, although for the detection of regions of interest (ROI) and with patches analyzed independently, a suitable increase in patch size (e.g., 960 × 960) can contribute to better accuracy. Additionally, averaging can occur at the regional level with regions classified as ROI and these then expanded over multiple patches (at increased risk of false negatives from missing small ROI).

Another study [5] focused on metastasis detection in breast cancer patients, proposing to automate the process of achieving accurate localization of tumors. By feeding DL with gigapixel images (100,000 × 100,000 pixels) the study found small tumors (100 × 100 pixels) in 92.4% of the cases. For the problem of differentiating cancer subtypes based on features observed at the image patch scale, it is ideal to consider discriminative patches for optimizing classification results (see, for instance, [6] about DL applications to glioma and lung cancer) that can match pathologists’ consensus. Finally, it is worth mentioning a radiomic study [7] for appropriate treatment assignment hypothesizing an association between local immune micro-environment features of non-small cell lung cancer (NSCLC) and patient outcomes. Based on immunohistochemical measures of programmed death ligand 1 (PDL1) expression and tumor-infiltrating lymphocytes, a quantitative assessment was made for two patient cohorts, after treating by surgical resection and extracting data from pretreatment CT imaging, and an immune pathology-informed model was built to cluster patients in relation to overall survival, which led to a radiomic signature.

It is worth a further note to mention radiopathomics, i.e., the combination of radiographic and digital pathology images. Recently proposed to better capture the hidden correlations between cancer phenotypes and tumor responses with the support of artificial intelligence (AI), investigating this direction might guide the clinicians into more individualized diagnosis, prognosis, and treatment for cancer patients.

#### 1.2.2. Biobanking

Connected with both pathology and radiology, the field of biobanking is also changing quickly (see [8] for a comprehensive review). Biobanking is especially expanding in parallel with the high-throughput computing developments designed to extract a wealth of quantitative features from bioimages derived from various acquisition technologies (CT, MR, PET, etc.). The expected result is to generate, and later validate, possible imaging biomarkers as a final product of marks, signals, and measurements that reflect novel disease phenotypes and quantifications that use novel types of data and metadata. The latter, once integrated within signatures obtained from combining risk and prognostic factors relevant at a clinical level and once joint with omics profiles, become useful for assessing pathophysiological conditions and response to treatment but also for enhancing patient management at a more personalized level.

#### 1.2.3. Radiology

In radiology, predictive modeling involves three concatenated steps, here summarized as (i) algorithmic treatment of tumor phenotypes translated into mineable features, (ii) detection of patterns explaining clinical outcomes, and (iii) association with endpoints. This process is computationally complex although efficiently solvable with ML algorithms. More importantly, it requires the integration of multilevel information (clinical and non) provided by interdisciplinary teams after suitable measurement standardization has occurred. Although necessary, validation of possible integrative signatures performed through unseen (given separation with training and testing sets) or external datasets can only mitigate the uncertainty linked to the inherent intratumor heterogeneity. This execution requires accurate partitioning to a variable resolution map aimed at maximal reproducibility, and calls for integration between the characterized imaging phenotypes and specific molecular marks, as in the case of radiogenomics.

In a recent study [9], various classification methods were tested to evaluate their predictive performance on a lung cancer dataset suitably split into training and validation subsets and were then assessed in terms of stability of feature selection (via resampling). Even if a ranking of methods appeared an important result of this work, the problem is that different imaging modalities and different cancers would likely induce changes in the ranking and/or suggest a better/worse performance of any specific method. Additionally, there might be a lack of balance in datasets as a typical example of bias generation. Blind application of ML algorithms is thus not sufficiently informative even if performed rigorously. Another approach was indicated in [10]. Here, a more dynamic perspective was considered by the fact that NSCLC features may change during therapy, for instance. These so-called delta-radiomics features were found to be particularly informative of tumor response, thus improving prognostic models. The limitation in this type of study is exactly the validation phase: This would require patient cohorts similarly screened by imaging during treatment, which can be solved necessarily by cross-validation analysis. Overall, the validity of the approach in a clinical setting remains only approximate because it is lacking the necessary calibration derived from matching predictions with observed outcomes [11].

It is clear from the described scenarios that ML and statistical methods need to deal with multiple and diverse data dimensions and parameters. To such objective difficulty, a main factor to be added is the need of validating once appropriate endpoints are defined based on the assessment of prognostic paths and therapy response. In such regards, prospective clinical trials are the ideal verification ground for radiomics [12]. In this perspective, the focus is on computational radiomics. Following a recent analysis [13] assessing the role of artificial intelligence targeted to precision oncology, among the identified challenges there were data multimodality and insufficiency jointly with the interpretability of ML predictive learning and its extensions. In an attempt to classify challenges specifically for radiomics, we can generalize such concepts with reference to a series of methodological approaches either currently in use or potentially usable.

## 2. Multimodality and Integrative Radiomics

In this section, three main aspects are considered in detail:

(a) Imaging multimodality, which combines imaging modalities to overcome the limitations of each single technique and augments the informative data volumes available to each pre-clinical experiment;
(b) Joint omics association, with a focus on genomic and metabolic aspects currently showing great promise for the discovery of new candidate imaging markers; and
(c) Role of features in radiomic models.

### 2.1. Inter-Modality Feature Integration Strategies

From a modeling standpoint, the fact that multimodal imaging features generally display very few similar associations with the underlying tumor characteristics offers concrete advantages. The least redundant and more independent the features are, the more predictive power the model can have. As each imaging modality performs at different spatial resolutions and voxel dimensions, a rationale for integrating potentially diverse information is naturally present. However, such a potential advantage comes with an important limitation, i.e., a large volume of samples would be needed to avoid false positive associations. As a second consideration, depending on the tumor type, the imaging modalities may complement each other across a variety of feature integration levels centered, for example, on phases, such as diagnosis, treatment, and patient follow-up. Thus, at one end, the modalities can scan and visualize the human body for diagnostic and treatment purposes or for understanding biological, physiological, and functional processes related to disease states (onset, progression, relapse, etc.), and at the other end, they may be useful for monitoring patients and assessing the effects from undergoing a treatment plan. Clearly, the data modeling strategy must adapt to the imaging developments while focusing on both the specific model parameterizations that the digital images allow and on the quantitative representation and characterization that are possible with new types of measurements. For cancer diagnostics, these steps tend to be highly influenced by the typical spatial heterogeneity and the imaging-related regional changes that alter the coarse-to-fine detail grid usually surrounding the anatomical structure.

### 2.2. Omics Associations

Radiogenomics [14,15,16,17] links radiomics with genomics by exploring their possible synergies, for instance, studying genotype variation together with the variability of response to treatment, or also the coupling between imaging phenotypes and gene expression patterns and signatures. Radiogenomics deals with both pathological and radiological aspects and often includes associations that bridge between the anatomic/histologic and genetic levels [8]. While radiogenomics is expected to better characterize tumor biology and its inherent heterogeneity (examples from glioblastoma multiforme [18,19], lung cancer [20,21], prostate cancer [22], and breast cancer [23,24,25]), a bottleneck occurs at the biomarker level. Here, it is hard to obtain consistency from the combined evidence types as this requires at the very minimum standardization operated at various levels. Biomarkers must be reproducible in order to be objective criteria for response assessment, also knowing that changes depend on spatiotemporal heterogeneity in part inherent to the tissue and in part occurring in the course of treatment [26].

At a metabolic level, early findings on PET radiomics are also available. Metabolic intratumor heterogeneity assessed thought images is based on the idea that it might inform on glucose metabolism, necrosis, oxygenation, vascularization, and angiogenesis, but a clear consensus on how to interpret the parameters has yet not been achieved. In a PET context, the characteristic trait is large voxel dimensions, which deliver quite approximate measurements in statistical terms, and this complication runs in parallel to a standardization phase that is particularly hard [27]. Overall, further motivation appears for integrating PET with other radiomic layers [28]. Interestingly, targeted studies have shown cancer sub-type signatures (in breast cancer, for instance) correlated with transcription factor expression [29].

### 2.3. Feature-Driven Model Selection

The recognized relevance of radiomics for precision medicine is due to the fact that it combines the tumor phenotype with individual variability. This multifaceted correlation requires ad hoc analysis and verification before objectively showing the ability to measure and organize a blend of characteristics that identify optimal medical intervention. The quantitative modeling aspects of radiomics address such a challenge by making systematic use of image data that are translated into detected features to be considered clinically useful when significant, i.e., prognostically or predictively reliable towards outcome parameters and endpoints (see also [30]). With many possible image features, the role played by DL is relevant, especially for image classification. In general, routine use in the clinical workflow would require an evaluation of the algorithmic performance across a variety of conditions and including all available annotations related to patient history and outcomes. Prognostic and predictive modeling imply different design strategies, quite evidently, and these eventually determine the achievable level of generalizability of biomarkers towards endpoints.

Generalizability and transferability of radiomic results are aspects of paramount importance that depend also on the model selection phase. Intuitively, the traits in the radiomic results that would deserve attention may depend on so many factors that both generalizability (different tumor type, for instance) and transferability (context shift due to differentiated conditions) appear as hurdles to be overcome. In guiding the effort of assimilating data from multiple streams, an integrative approach should be particularly informative about aspects, such as early detection, tumor evolution, metastatic patterns, acquired resistance, recurrence, etc. Finding their marks and descriptors lies at the core of quantitative radiomics that operates over relevant (i.e., significant and reproducible) features.

## 3. Learning Approaches and Significance for Radiomics

This section discusses a few types of learning approaches by introducing them and analyzing their principled role and utilization in radiomics. Significance is discussed through some selected application examples. Although priority is assigned to ML and DL, also other learning techniques are described in both current and expected impacts.

### 3.1. Machine Learning (ML)

#### 3.1.1. Definition

ML is mostly aimed to learn data and image features and generate class labels that allow segmentation or classification in either a supervised or unsupervised way. The focus is on modeling probabilistically the input x-labels’ y transform, which in the radiomic context links the intensity values characterizing the radiological images to the underlying tissue types. ML is instrumental to extracting many quantitative features in a (semi-) automated way to allow complex detection tasks, such as identifying patterns that are hard to interpret or discovering markers and signatures relevant to the disease course and the prediction of the response to therapy [31]. ML algorithms characterize data with features from various types of scan images referring to a certain region of interest (ROI) and such features can inform of intra-tumor heterogeneity by describing voxel intensities, shapes, edges, and textures [32].

#### 3.1.2. Significance

In a study on lung cancer aimed at predicting survival [33], an analysis of variability was performed indicating that the choice of the classifier is the most influential decision with regards to performance variation, explaining about one third. In another study [34], the radiomic phenotypes extracted from MRI classified five molecular glioma subtypes, achieving almost an 82–90% accuracy depending on the absence or presence of histology diagnostic information, respectively. An important point to stress is that informative features in radiomic analysis can be of different types, semantic and non-semantic, with a variable degree of quantitative descriptions together with those treated in an automated way and more naturally and specifically linked to clinical outcomes [35]. In general, more traditional ML methods are efficient but appear somehow limited regarding the scalability of the algorithms, which partially explains the tendency of associating radiomic applications to DL approaches. Another current gap impacting clinical radiotherapy points to the need of adapting to the typically complex dynamics of decision-making processes characterized by underreported events or missing data that might affect the predictive role of some model drivers.

### 3.2. Deep Learning (DL)

#### 3.2.1. Definition

There is a quite diffuse consensus about the efficiency and reliability of DL as an inference tool in medical imaging and radiomics. First, being dimensionless, it can work well with big data. Second, differently from ML algorithms, DL performs more directly in both a generative way (adversarial networks, variational autoencoders, etc.) and discriminative way (convolutional neural networks etc.). For instance, it is possible to use DL algorithms, such as convolutional neural networks, to efficiently replace the feature selection task operated in image segmentation. The advantage offered is preservation of spatial information, and indeed once the image has been accurately segmented all the information remains within the network. This allows the image features to be directly extracted with no extra errors introduced into radiomic analyses because of feature calculations [36].

#### 3.2.2. Significance

DL offers a major potential for a series of tasks, such as facilitating and/or improving lesion detection, enabling accurate differential diagnoses, assessing treatment effects, and finally providing better patient stratification and prognostic paths. Among many DL-driven publications covering cancer classification tasks, we recall two very recent studies centered on lymph node metastasis as a significant prognostic factor whose accurate prediction is key for optimizing treatment in patients with head and neck and lung cancers, respectively. The former [37] presents a many-objective model with a 3-D convolutional network exploiting spatial information for the classification of normal and diseased nodes together with suspicious ones, and it achieves predictive gains compared to using PET and CT. The latter [38] evaluates lymph node status in >500 early lung cancer patients with preoperative CT demonstrating significant performance gain in prediction accuracy of metastasis by using cross-modal 3-D-DL integrating CT scan and clinical information.

### 3.3. Reinforcement Learning (RL)

#### 3.3.1. Definition

RL [39] leverages the idea that an optimal action facing unknown environmental states (assumed to undergo Markov dynamics) is associated to a reward. Therefore, a learner acts by maximizing the pay-off. RL is a goal-oriented strategy that essentially maximizes reward over multiple actions contributing to it. Scale is the problem, with real-world domains involving many decisions and the pay-off being better defined when only a small set of decisions can be selected in terms of relevance. RL biomedical problems focus on finding optimal treatments for patients and often involve drugs. At a computational level, a Markov decision process (MDP) is formulated and a cost function is associated to the model to find an optimal trajectory of states and actions (e.g., optimal control) concerning patient management.

#### 3.3.2. Significance

Deep RL is a possible model framework and was analyzed in [40], as an example, based on historical treatment plans and with the scope of finding feasible strategies for automating the radiation adaptation protocols of dose escalation in NSCLS patients. Specifically, the agent here interacts with a radiotherapy artificial environment (RAE) reconstructed by a so-called transitional network. Compared to clinician choices, automated dose adaptation by deep RL showed results similar to those obtained by clinicians. There are also other applications with a focus on detection and treatment response prediction [41], like CT detection of pulmonary nodules for lung cancer screening in 590 persons (see [42]). Here, in particular, the good performance measures were based on raw CT images analyzed through states and classifying nodules as present or not, thus supporting decisions about the opportunity of follow-up tests and related expenditures saving.

Putting it into perspective, how to cast RL within a transfer learning (TL) framework, i.e., allowing generalization to occur across tasks, is a very relevant problem. This was studied with rewards variable between tasks but within a fixed environment’s dynamics [43]. The idea that rewards may be defined to induce hierarchical task decomposition with a task generating either independent or temporally dependent subtasks impacts radiomics for at least two reasons. First, building a predictive model from the extracted features is functional to attaining a radiomic pattern or signature. The tasks defining prediction can feed models enabling more or less independent relationships, say classification versus survival analysis, and produce prediction scores involving suitably combined signatures. Second, the interpretability of such predictive models involves macro- and micro-analysis of tasks ranging from feature selection quality to relative importance of ROI voxels associated to predicted outcomes.

The two next related topics, value learning and Q-learning, are integrated with RL model strategies and suggest considerations of potentially high impact for radiomics.

### 3.4. Value-Based RL (VL)

#### 3.4.1. Definition

The problem of value estimation is RL refers to learning the long-term consequences of being in a certain state. Naturally enough, there is uncertainty that makes it essential to estimate the value. A strategy is based on identifying a value function and measuring the total rewards expected from a particular state following a specific policy. Policy iteration occurs when the policy undergoes repeatedly evaluation and refinement till improvement assessed via the value function reaches optimality. This requires either an adaptive model to change the value function for the states or an automatic learner (like DL) to reach an approximate solution. The data burden increases due to the scoring of actions in each state, which requires computation of the value function to measure the expected action-associated rewards. VL has been discussed in general and technical terms by [44,45,46], among others.

#### 3.4.2. Significance

Currently, and to the best of our knowledge, the role of VL in RL applications proposed in radiomics has been not so central. We see a major potential role in supporting radiomic decision processes, for instance, when a learner clinician wants to exploit a set of actions or utility functions in a probabilistic way, i.e., ranging with certain probabilities assigned across values and preferences. As a practical example, an important aspect refers to considering maximal utility corresponding to limited risk inherent to some actions, for instance, those functional to goals fixed within radiotherapy workflows.

### 3.5. Q-Learning (QL)

#### 3.5.1. Definition

QL [47] solves the problem of learning a value function by a strategy that finds an optimal policy given an MDP with a function defined as the average discounted sum of rewards expected in the future steps and moving from the current state. Knowing the expected reward of each action at every step corresponds to knowing the sequence of actions to be performed for eventually generating the maximum total reward. The expectation involves computing all possible paths starting from current states and covering all possible future benefits, given a discounting factor to trade-] off the importance of immediate versus future rewards.

Finding the optimal function requires the agent to try repeatedly each action in every state, but QL does not specify the actions that an agent should take at each state. Although this process may lead to using DL, this is not considered computationally attractive and other solutions can be identified, such as a policy gradient, which makes actions with better rewards more likely. QL simply aims to preserve the best estimate of states’ values by constructing a value function on the state space and updating it according to an optimal choice of action at the following state.

#### 3.5.2. Significance

Of interest for applications are a couple of considerations about the role of temporality, which is central to radiomics. Differently from RL that optimizes averages rewards (using equal weights), QL optimizes discounted rewards by assigning superior weights to near-term ones. This potentially impacts profiling and prognostication in hybrid radiomic approaches that try to exploit multiple data sources and improve predictive scores from their fusion. This strategy involves a comprehensive process and integrated framework in which sequences of decisions and recommendations must adapt to the evolving health trajectory of treated patients. Such dynamic treatment regimens define treatment processes as sequences of decision rules and guide clinicians to treat patients over time more interactively according to personalized solutions rather than aiming at the most favorable clinical outcome on average, and still be considered near-optimal, i.e., achieving maximal expected outcomes when applied to stratified patient populations.

### 3.6. Active Learning (AL)

#### 3.6.1. Definition

The task of AL [48] is primarily iterative selection, i.e., finding what data allow learning of the model once this becomes annotated (labeling action). Collecting labels in medical imaging can be an expensive process requiring special expertise. AL allows the training of classifiers with associated low-annotation costs as it predicts which unlabeled instances should be labeled. This learning strategy can be combined with RL to learn an active learner [49], or can be made data-driven by setting a regression for the prediction of the error reduction for a sample in a certain state [50], and finally can also be made transferable across datasets to improve AL via regularization [51]. When the focus is on fixing criteria that generalize across datasets, an interesting solution is to define a policy parameterized by a dataset embedding [52,53]. This means that an auxiliary network predicts weights for a target network whose input dimensionality is handled by a reduced number of parameters [53]. As an important note, by interpreting the AL criterion as a deep RL problem, one can get the optimal AL policy (i.e., a network parameterized by weights).

#### 3.6.2. Significance

An obvious criterion for using AL in radiomics or not relies on the evaluation of the capacity that the features have to be mutually informative or adding separate value as this can lead to increased prediction power. Therefore, given suitably selected patients and available imaging modalities, discovery requires a sequence of steps leveraging initially standardization of image acquisition protocols, pre-processing, and segmentation, and then feature extraction and selection. However, Sharma et al. [54] noticed how DL requires large annotated data for training well and thus enabled accurate segmentation with reduced labelled data by combining DL with AL in order to find points from the unlabeled samples and select the most uncertain. Zhou et al. [55] presented a method with active and incremental fine-tuning attributes designed to integrate AL and TL into a model that first pre-trains CNN for selecting unannotated samples for annotation, and then performs iterative tuning via newly annotated samples in order to improve the overall performance incrementally.

To conclude this section, we emphasize a major impact area where the above methodologies are expected to exert influence in the future, i.e., data-driven decision support systems. In the general healthcare field, these may allow clinicians to deliver better personalized treatments. In such a regard, both DL and deep RL have been only marginally exploited (see [56] and an application on bone transplant registry data). A first survey on the topic was recently published [57], with challenges mainly identified in (a) data deficiency and intervention variations, which can both make the learned policies sub-optimal; (b) lack of strategies to find appropriate states, actions, and reward functions, in particular those able to balance the trade-off between short- and long-term success; and (c) an absence of performance benchmarks due to the limited availability of applications.

To such current bottlenecks we also add another one: Human interactions within complex environments occur non-synchronously. A recent study [58] proposed deep RL of marked temporal point processes to characterize actions from agents and feedback from the environment seen as asynchronous stochastic discrete events. This has potential utility in radiotherapy where operations run continuously and induce periodic progress reports, incremental results, or state changes, but also for applications in distributed radiomics (see the multi-center study in [59] centered on a radiomic signature developed at one site and validated in its performance at another site).

**Figure 1:**
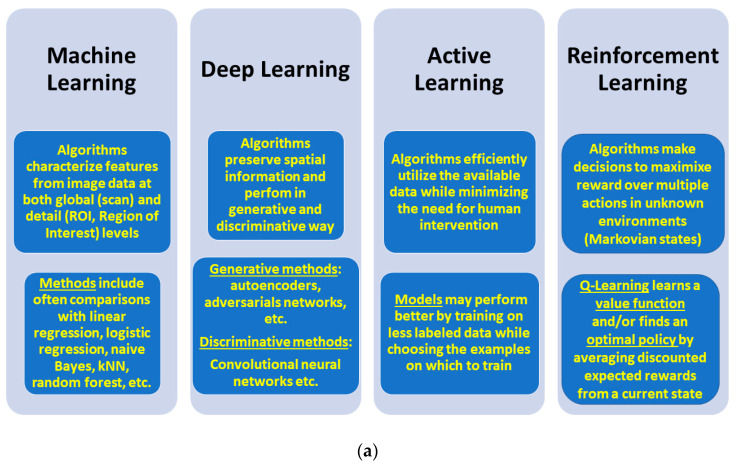

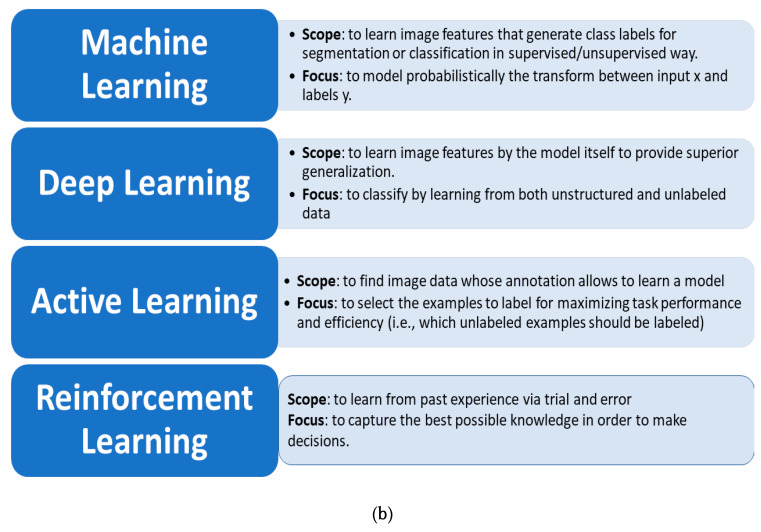
Salient information about learning techniques. Methodological aspects (**a**, top panel); Scope and focus (**b**, bottom panel).

**Figure 2:**
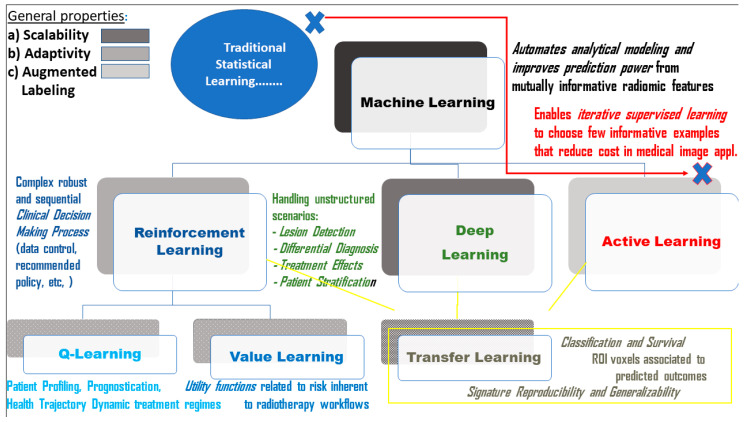
Significance of learning for Radiomics. General properties (gradients boxes) and distinct characteristics (floating text). Note: the aggregate black color assigned to ML is due to multiple properties.

Figure 1 summarizes all of the above-listed learning techniques and covers the salient methodological aspects that characterize them (top panel), together with scope and focus (bottom panel). Figure 2 emphasizes general and distinct properties of these learning techniques (including TL) with reference to their significance for radiomics.

## 4. Application Contexts for Radiomics

Some of the salient applications with reference to specific impact areas and disciplines are summarized below in Table 1, with special emphasis assigned to the learning modalities involved.

Radiomics spans various clinical domains in terms of modeling treatment risk [76,77], improving diagnosis [78,79], predicting treatment outcomes [80,81], and toxicity [82,83].

*Dermatology:* It is very challenging for trained dermatologists to interpret and diagnose skin lesions due to their large variability in sizes, shades, and textures. Yet, trained with massive annotated images, the CNN has achieved dermatologist-level accuracy in classifying the various types of skin lesions [60]. The integration of advanced DL algorithms with mobile technology will offer radically new solutions for early cancer detection by providing highly accurate diagnostic capabilities in a cost-effective manner, benefiting millions of people around the world. Recent radiomics applications have also appeared [84,85].

*Ophthalmology:* As the diabetic retinopathy is quite prevalent (18–28.5%) among individuals with diabetes, most guidelines recommend annual screening for those with no or mild diabetic retinopathy, repeat examination in 6 months for moderate diabetic retinopathy, and an ophthalmologist evaluation for severe diabetic retinopathy. While manual interpretation of retinal photography is a widely accepted screening tool for diabetic retinopathy, automated grading of diabetic retinopathy can help to increase the efficiency and reproducibility and improve patient outcomes by providing early detection and treatment. Based on an Inception-v3 architecture [86], researchers from Google developed a CNN that can detect diabetic retinopathy in retinal fundus photographs with high sensitivity (>87%) and specificity (>98%) [61]. Another commonly used imaging modality in ophthalmology is optical coherence tomography (OCT), which is often used in diagnosing age-related macular degeneration (AMD), a common eye condition and a leading cause of vision loss among people aged 50 and older. Similar to fundus photography, a CNN ensemble has been developed to automatically segment and quantify the OCT images, improving prognosis and management of macular diseases [62]. Radiomic applications have recently appeared too [87,88].

*Pathology:* Accurate and efficient interpretation of the hematoxylin and eosin (H&E) slide has remained the core function of pathologists for many years. Yet, the large variation in imaging hardware, slide preparation, magnification, and staining techniques has made the quantification of slide images quite challenging [63]. DL technologies represent new instruments to help pathologists extract unprecedented and colossal amounts of objective and multiparametric morphologic information, which is important in the accurate diagnosis of many types of cancers [64]. The coupling of AI-assisted interpretation and pathologists’ oversee and approval will be vital for successful implementation of precision oncology in the near future [65].

*Radiation Oncology:* This clinical field is uniquely positioned to harness the power of big data as vast amounts of data are generated at an unprecedented pace for individual patients in imaging studies and radiation treatments worldwide [89]. A large portion of patient big data include the anatomical and functional information from diagnostic and therapeutic imaging modalities, such as CT, PET, MRI, and cone-beam CT (CBCT). Radiomics is now increasingly integrated within clinical decision processes and consistently used in automatic segmentation of the tumor and organ volumes, assessment of treatment response, prediction of patient outcomes, and evaluation of post-treatment toxicity [66,67,68]. In parallel, it is expected that learning approaches will be increasingly adopted and augmented in their ability to merge qualitative and quantitative components beyond standard ML and DL.

*Brain Imaging:* CT, PET, MRI, and functional MRI (fMRI) images yield radiomic data that characterize the brain tissues and tumors in terms of structures, textures, malignancy, and metastasis and contribute to diagnostic and prognostic predictions for individual patients [69,70,71]. Here, the complement offered to ML and DL approaches by mechanistic models is relevant and will most likely consolidate into integrated learning solutions.

*Thoracic Imaging:* Lung cancer is one of the most common and deadly tumors, and while the targeted screening with low-dose CT or MRI helps identify pulmonary nodules whose early detection can save many patient lives, radiomics of these images can automatically identify the nodules and categorize them as either benign or malignant [72,73]. RDL and variants often applied as preferred inference approaches are destined to be increasingly refined.

*Breast Imaging:* Mammography has been widely used for breast cancer screening. However, it is technically challenging to interpret the mammography images, due to the large variations in breast tissue texture, density, and presence of small deposits of calcium in the breast. DL-driven radiomics of mammography images can continue to assist in interpreting, identifying, and characterizing the cancerous breast tissues for early detection and intervention [74].

*Abdominal Imaging:* Radiomics based on colonoscopy images has been found to be very effective in detecting and classifying malignant polyps [75]. The American Cancer Society (ACS) recommends that people at average risk of colorectal cancer start regular screening with colonoscopy at age of 45. This is because colonic polyps that are undetected or misclassified pose a potential risk of colorectal cancer. Although most polyps are initially benign, they can become malignant over time. Hence, early detection and consistent monitoring with robust AI-based tools are critical and their implementation will consequently feed predictive ML approaches.

## 5. Discussion

The final notes are dedicated to some of the lessons learned and some of the current bottlenecks.

First, unlike other omics disciplines, radiomics directly deals with spatiotemporal heterogeneity. The various combinations of multimodal imaging and the possible omics associations offer great opportunities to add value to the analyses that radiomics typically targets to the possible discovery of biomarkers and the design of highly integrated clinical decision support systems [90]. Second, an important limitation refers to clinical trials and concerns the risk incurred by newly generated biomarkers regarding both experimental and imaging inconsistency. Therefore, suitable standardization criteria, analytical approaches, and trial design are required [11,27]. In particular, prospective clinical trials may be expected to take advantage from learning improvements in terms of treatment adaptation and refined patient stratification [12]. Third, the classification of tumors in subtypes based on imaging phenotypes [91] (jointly with molecular features) is gaining importance together with the role that a superior quality tumor partitioning may play to allow sophisticated image phenotyping (intratumor subregion characterization) [4,92].

At a methodological level, a fourth aspect concerns DL and the need of opening the black box to allow better interpretation, reproducibility, and generalizability, something now gaining extensive attention by mathematicians moving from fragmented to consensus model solutions. This implies that while contexts remain critical for the quality and informativeness of specific features, the goal is to control that redundancies and complexities coming from both technical and biological artifacts do not interfere or prevail [93]. At one end, this change will reflect further relaxation of the one-model-fits-all strategy and stimulate recourse to TL toward improved predictive performance [94]. At another end, new methods will emerge to provide better representations for the encoded inputs via concepts like networks deconvolution, inversion, and dissection, among others (see [95]). Fifth and last, in order to face the challenge of intratumor heterogeneity, the quantification of tumor abundance at the voxel level is becoming an important direction in response assessment and recurrence risk studies [4,95,96,97]. This might help the identification of subregions, for instance, those metabolically active and defined as high risk [92], and may also inspire strategy to mitigate the effects of unbalanced data (for instance, when an outcome is over-represented) and thus decisional bias [12].

Finally, it is worth mentioning that recent studies reported ML-driven radiopathomics applications (e.g., SVM, logistic regression) for prognosis of glioblastoma [98] and grading of glioma [99], prediction of pathologic response in the locally advanced rectal cancer (LARC) [100], diagnosis of lung nodule subtypes [101], and detection of high-grade prostate cancer tumors missed by radiologists [102]. Model performance ranged from 0.8 to 0.9 in the accuracy, sensitivity, specificity, and AUC. Due to higher discrimination power compared to radiographic images or pathology images alone, it may be reasonably expected that radiopathomics will be playing a relevant role in the diagnosis, prognosis, and treatment assessment for individual cancer patients, thus justifying its contributions in coordinated efforts on the clinical trial [103] and public sharing of research resources [104].

## 6. Concluding Remarks

The learning techniques that were presented in this perspective include only part of the methods and approaches that are available but share the main challenges usually faced in applications. The current focus is on the need of reconciling radiomic features retrieved from multiple imaging modalities and on integrating a variety of feature types aimed at providing improved predictive learning for specific targets. A radiomic analysis is valuable depending on the information carried by the imaging datasets and becomes medically significant when enhanced information can be obtained by correlations with clinical outcome data. Then, the modeling component plays a central role to guarantee the most effective amalgamation of evidence types and context variables toward optimal feature selection.

Clinical decisions that account for radiomic information are determined, among other factors, by volumes of heterogeneous data for which the centrality of learning algorithmically is destined to grow. For instance, the adoption of DRL techniques finds clear utility in problems, such as optimization of patients’ medication choice and dosage. Temporality is a driver of learning therefore and radiomic modeling ultimately depends on the ability to acquire imaging data and extract features at different times and patient-specific contexts to assess longitudinally the value of health records. Only the flexibility allowed by model solutions regularly updated and accurately validated will ensure that results and scores can be used for predictions impacting disease biomarkers, therapy assessments, and patients’ stratifications.

The advent of EHR offers an opportunity to build data resources connecting patient data and histories with genetics, digitized medical images, and treatment outcomes, thus triggering the use of learning techniques in full integrated modality (see, for instance, [105]). In turn, challenging problems related to data heterogeneity, scale, and feature types will appear and induce a revision of statistical and ML paradigms, such as dimension reduction and data fusion. Finally, causality, interpretability, and generalizability will also need to be newly prioritized in view of next-generation learning techniques.

## Figures and Tables

**Table 1:** Applications in clinical domains: significance for medical imaging and radiomics.

Clinical Domains	Modalities	Computational Approaches and Methods	Top Performance Achieved	Ref.
*Dermatology*	Skin lesion images	DL—CNN	AUC 0.94–0.96	[60]
*Ophthalmology*	Fundus photography	DL—CNN	Sensitivity 0.97 Specificity 0.93	[61]
	Optical coherence tomography	DL—CNN	AUC 0.97 Sensitivity 0.90	[62]
*Pathology*	Histopathologic images	Random Forest, SVM, CNN	PPV 0.94, NPV 0.92, F1 0.91	[63,64,65]
*Radiation Oncology*	CT/CBCT	CNN, Distributed DNN	DSC 0.81	[66]
	MRI	CNN, ANN	AUC 0.86	[67]
	PET	SVM, KNN	AUC 0.95 Sensitivity 0.95 Specificity 0.95	[68]
*Brain Imaging*	CT	CNN	AUC 0.90–0.96	[69]
	MRI/fMRI	Stacked auto-encoders, deep Boltzmann machines, DNN, CNN	Sensitivity 0.93 Specificity 0.82	[70]
	PET	Autoencoder, CNN	AUC 0.74–0.90	[71]
*Thoracic Imaging*	CT	CNN	AUC 0.94	[72]
	MRI	CNN, RNN	Dice coefficient 0.80	[73]
*Breast Imaging*	Mammography	CNN	AUC 0.98 Sensitivity 0.86 Specificity 0.96	[74]
*Abdominal Imaging*	Colonoscopy	CNN	AUC 0.99 Accuracy 0.96	[75]

Notes: acronyms used for the methods appear according to standard literature.

## References

[1] Song J., Yin Y., Wang H., Chang Z., Liu Z., Cui L. (2020). A review of original articles published in the emerging field of radiomics. Eur. J. Radiol..

[2] Hägele M., Seegerer P., Lapuschkin S., Bockmayr M., Samek W., Klauschen F., Müller K.R., Binder A. (2020). Resolving challenges in deep learning-based analyses of histopathological images using explanation methods. Sci. Rep..

[3] Xia T., Kumar A., Feng D., Kim J. Patch-level tumor classification in digital histopathology images with domain adapted deep learning. Proceedings of the 2018 40th Annual International Conference of the IEEE Engineering in Medicine and Biology Society (EMBC).

[4] Beaumont J., Acosta O., Devillers A., Palard-Novello X., Chajon E., de Crevoisier R., Castelli J. (2019). Voxel-based Identification of Local Recurrence Sub-Regions from Pre-Treatment PET/CT for Locally Advanced Head and Neck Cancers. EJNMMI Res..

[5] Liu Y., Gadepalli K., Norouzi M., Dahl G.E., Kohlberger T., Boyko E., Venugopalan S., Timofeev A., Nelson P.Q., Corrado G.S. (2017). Detecting Cancer Metastases on Gigapixel Pathology Images. arXiv.

[6] Hou L., Samaras D., Kurc T.M., Gao Y., Davis J.E., Saltz J.H. Patch-based convolutional neural network for whole slide tissue image classification. Proceedings of the IEEE conference on computer vision and pattern recognition.

[7] Tang C., Hobbs B., Amer A., Li X., Behrens C., Rodriguez Canales J., Parra Cuentas E., Villalobos P., Fried D., Chang J.Y. (2018). Development of an immune-pathology informed radiomics model for non-small cell lung cancer. Sci. Rep..

[8] Coppola L., Cianflone A., Grimaldi A.M., Incoronato M.R., Bevilacqua P., Messina F., Baselice S., Soricelli A., Mirabelli P., Salvatore M. (2019). Biobanking in health care: Evolution and future directions. J. Transl. Med..

[9] Parmar C., Grossmann P., Rietveld D., Rietbergen M.M., Lambin P., Aerts H.J. (2015). Radiomic Machine-Learning Classifiers for Prognostic Biomarkers of Head and Neck Cancer. Front. Oncol..

[10] Fave X., Zhang L., Yang J., Mackin D., Balter P., Gomez D., Followill D., Jones A.K., Stingo F., Liao Z. (2017). Delta-radiomics features for the prediction of patient outcomes in non-small cell lung cancer. Sci. Rep..

[11] Lambin P., Leijenaar R.T.H., Deist T.M., Peerlings J., de Jong E.E.C., van Timmeren J., Sanduleanu S., Larue R.T.H.M., Even A.J.G., Jochems A. (2017). Radiomics: The bridge between medical imaging and personalized medicine. Nat. Rev. Clin. Oncol..

[12] Peeken J.C., Bernhofer M., Wiestler B., Goldberg T., Cremers D., Rost B., Wilkens J.J., Combs S.E., Nüsslin F. (2018). Radiomics in radiooncology-challenging the medical physicist. Phys. Med..

[13] Azuaje F. (2019). Artificial intelligence for precision oncology: Beyond patient stratification. NPJ Prec. Onc..

[14] Rutman A.M., Kuo M.D. (2009). Radiogenomics: Creating a link between molecular diagnostics and diagnostic imaging. Eur. J. Radiol..

[15] Kuo M.D., Jamshidi N. (2014). Behind the numbers: Decoding molecular phenotypes with radiogenomics—Guiding principles and technical considerations. Radiology.

[16] Rosenstein B.S., West C.M., Bentzen S.M., Alsner J., Andreassen C.N., Azria D., Barnett G.C., Baumann M., Burnet N., Chang-Claude J. (2014). Radiogenomics: Radiobiology enters the era of big data and team science. Int. J. Radiat. Oncol. Biol. Phys..

[17] West C., Rosenstein B.S. (2010). Establishment of a radiogenomics consortium. Int. J. Radiother. Oncol..

[18] Kickingereder P., Bonekamp D., Nowosielski M., Kratz A., Sill M., Burth S., Wick A., Eidel O., Schlemmer H.-P., Radbruch A. (2016). Radiogenomics of glioblastoma: Machine learning-based classification of molecular characteristics by using multiparametric and multiregional MR imaging features. Radiology.

[19] Hong E.K., Choi S.H., Shin D.J., Jo S.W., Yoo R.-E., Kang K.M., Yun T.J., Kim J.-H., Sohn C.H., Park S.-H. (2018). Radiogenomics correlation between MR imaging features and major genetic profiles in Glioblastoma. Eur Radiol..

[20] Thawani R., McLane M., Beig N., Ghose S., Prasanna P., Velcheti V., Madabhushi A. (2018). Radiomics and radiogenomics in lung cancer: A review for the clinician. Lung Cancer.

[21] Das A.K., Bell M.H., Nirodi C.S., Story M.D., Minna J.D. (2010). Radiogenomics predicting tumor responses to radiotherapy in lung cancer. Semin. Radiat. Oncol..

[22] Stoyanova R., Takhar M., Tschudi Y., Ford J.C., Solórzano G., Erho N., Balagurunathan Y., Punnen S., Davicioni E., Gillies R.J. (2016). Prostate cancer radiomics and the promise of radiogenomics. Transl. Cancer Res..

[23] Pinker K., Chin J., Melsaether A.A., Morris E.E., Moy L. (2018). Precision medicine and radiogenomics in breast cancer: New approaches toward diagnosis and treatment. Radiology.

[24] Pang T., Wong J.H.D., Ng W.L., Chan C.S. (2020). Deep learning radiomics in breast cancer with different modalities: Overview and future. Exp. Syst. Appl..

[25] Saha A., Harowicz M.R., Mazurowski M.A. (2018). Breast cancer MRI radiomics: An overview of algorithmic features and impact of inter-reader variability in annotating tumors. Med. Phys..

[26] Bi W.L., Hosny A., Schabath M.B., Giger M.L., Birkbak N.J., Mehrtash A., Allison T., Arnaout O., Abbosh C., Dunn I.F. (2019). Artificial intelligence in cancer imaging: Clinical challenges and applications. CA Cancer J. Clin..

[27] Cook G.J.R., Azad G., Owczarczyk K., Siddique M., Goh V. (2018). Challenges and promises of PET radiomics. Int. J. Radiat. Oncol. Biol. Phys..

[28] Ha S., Park S., Bang J.I., Kim E.K., Lee H.Y. (2017). Metabolic radiomics for pretreatment 18F-FDG PET/CT to characterize locally advanced breast cancer: Histopathologic characteristics, response to neoadjuvant chemotherapy, and prognosis. Sci. Rep..

[29] Palaskas N., Larson S.M., Schultz N., Komisopoulou E., Wong J., Rohle D., Campos C., Yannuzzi N., Osborne J.R., Linkov I. (2011). 18F-fluorodeoxy-glucose positron emission tomography marks MYC-overexpressing human basal-like breast cancers. Cancer Res..

[30] Katsila T., Matsoukas M.T., Patrinos G.P., Kardamakis D. (2017). Pharmacometabolomics informs quantitative radiomics for glioblastoma diagnostic innovation. OMICS.

[31] Langs G., Röhrich S., Hofmanninger J., Prayer F., Pan J., Herold. C., Prosch H. (2018). Machine learning: From radiomics to discovery and routine. Radiologe.

[32] Giraud P., Giraud P., Gasnier A., El Ayachy R., Kreps S., Foy J.-P., Durdux C., Huguet F., Burgun A., Bibault J.-E. (2019). Radiomics and machine learning for radiotherapy in head and neck cancers. Front. Oncol..

[33] Parmar C., Grossmann P., Bussink J., Lambin P., Aerts H.J.W.L. (2015). Machine learning methods for quantitative radiomic biomarkers. Sci. Rep..

[34] Lu C.F., Hsu F.T., Hsieh K.L., Kao Y.-C.J., Cheng S.-J., Hsu J.B.-K., Tsai P.-H., Chen R.-J., Huang C.-C., Yen Y. (2018). Machine learning-based radiomics for molecular subtyping of gliomas. Clin. Cancer Res..

[35] Liu Z., Feng B., Li C., Chen Y., Chen Q., Li X., Guan J., Chen X., Cui E., Li R. (2019). Preoperative prediction of lymphovascular invasion in invasive breast cancer with dynamic contrast-enhanced-MRI-based radiomics. J. Magn. Reson. Imaging..

[36] Li Q., Bai H., Chen Y., Sun Q., Liu L., Zhou S., Wang G., Liang C., Li Z.-C. (2017). A fully-automatic multiparametric radiomics model: Towards reproducible and prognostic imaging signature for prediction of overall survival in glioblastoma multiforme. Sci. Rep..

[37] Chen L., Zhou Z., Sher D., Zhang Q., Shah J., Pham N.-L., Jiang S., Wang J. (2019). Combining many-objective radiomics and 3D convolutional neural network through evidential reasoning to predict lymph node metastasis in head and neck cancer. Phys. Med. Biol..

[38] Zhao X., Wang X., Xia W., Li Q., Zhou L., Li Q., Zhang R., Cai J., Jian J., Fan L. (2020). A cross-modal 3D deep learning for accurate lymph node metastasis prediction in clinical stage T1 lung adenocarcinoma. Lung Cancer.

[39] Thrun S., Schwartz A. (1995). Finding Structure in Reinforcement Learning.

[40] Tseng H.H., Luo Y., Cui S., Chien J.T., Ten Haken R.K., Naqa I.E. (2017). Deep reinforcement learning for automated radiation adaptation in lung cancer. Med. Phys..

[41] Ghesu F.C., Krubasik E., Georgescu B., Singh V., Zheng Y., Hornegger Y., Comaniciu D. (2016). Marginal space deep learning: Efficient architecture for volumetric image parsing. IEEE Trans. Med. Imaging.

[42] Ali I., Hart G.R., Gunabushanam G., Liang Y., Muhammad W., Nartowt B., Kane M., Ma X., Deng J. (2018). Lung nodule detection via deep reinforcement learning. Front. Oncol..

[43] Barreto A., Dabney W., Munos R., Hunt J., Schaul T., van Hasselt H., Silver D. (2017). Successor Features for Transfer in Reinforcement Learning.

[44] Corrêa N.K., de Oliveira N. (2020). Dynamic models applied to value learning in artificial intelligence. arXiv.

[45] Everitt T., Hutter M. (2016). Avoiding wireheading with value reinforcement learning. Artif. Gen. Intellig..

[46] Leike J., Krueger D., Everitt T., Martic M., Maini V., Legg S. (2018). Scalable agent alignment via reward modeling: A research direction. arXiv.

[47] Watkins C.J.C.H., Dayan P. (1992). Q-learning. Mach. Learn..

[48] Buchman P., Sordoni A., Trischler A. (2017). Learning algorithms for active learning. arXiv.

[49] Woodward M., Finn C. (2017). Active one-shot learning. arXiv.

[50] Konyushkova K., Sznitman R., Fua P. (2017). Learning active learning from data. Adv. NIPS.

[51] Chu H.-M., Lin H.-T. (2020). Can active learning experience be transferred?. arXiv.

[52] Pang K., Dong M., Wu Y., Hospedales T. (2018). Meta-learning transferable active learning policies by deep reinforcement learning. arXiv.

[53] Romero A., Carrier P.L., Erraqabi A., Sylvain T., Auvolat A., Dejoie E., Legault M.-A., Dubé M.-P., Hussin J.G., Bengio Y. (2017). Diet networks: Thin parameters for fat genomics. arXiv.

[54] Sharma D., Shanis Z., Reddy C.K., Gerber S., Enquobahrie A. (2019). Active learning technique for multimodal brain tumor segmentation using limited labeled images. Domain Adaptation and Representation Transfer and Medical Image Learning with Less Labels and Imperfect Data.

[55] Zhou Z., Shin J.Y., Gurudu S.R., Gotway M.B., Liang J. (2018). AFT*: Integrating Active Learning and Transfer Learning to Reduce Annotation Efforts. arXiv.

[56] Liu Y., Logan B., Liu N., Xu Z., Tang J., Wang Y. Deep reinforcement learning for dynamic treatment regimes on medical registry data. Proceedings of the 2017 IEEE International Conference on Healthcare Informatics.

[57] Liu S., Ngiam K.Y., Feng M. (2019). Deep reinforcement learning for clinical decision support: A brief survey. arXiv.

[58] Upadhyay U., De A., Gomez-Rodriguez M. (2018). Deep Reinforcement Learning of Marked Temporal Point Processes. arXiv.

[59] Shi Z., Zhovannik I., Traverso A., Dankers F., Deist T.M., Kalendralis P., Monshouwer R., Bussink J., Fijten R., Aerts H. (2019). Distributed radiomics as a signature validation study using the Personal Health Train infrastructure. Sci. Data.

[60] Esteva A., Kuprel B., Novoa R.A., Ko J., Swetter S.M., Blau H.M., Thrun S. (2017). Dermatologist-level classification of skin cancer with deep neural networks. Nature.

[61] Gulshan V., Peng L., Coram M., Stumpe M.C., Wu D., Narayanaswamy A., Venugopalan S., Widner K., Madams T., Cuadros J. (2016). Development and validation of a deep learning algorithm for detection of diabetic retinopathy in retinal fundus photographs. JAMA.

[62] Orlando J.I., Gerendas B.S., Riedl S., Grechenig C., Breger A., Ehler M., Waldstein S.M., Bogunović H., Schmidt-Erfurth U. (2020). Automated quantification of photoreceptor alteration in macular disease using optical coherence tomography and deep learning. Sci. Rep..

[63] Gurcan M.N., Boucheron L.E., Can A., Madabhushi A., Rajpoot N.M., Yener B. (2009). Histopathological image analysis: A review. IEEE Rev. Biomed. Eng..

[64] Komura D., Ishikawa S. (2018). Machine learning methods for histopathological image analysis. Comput. Struct. Biotechnol. J..

[65] Djuric U., Zadeh G., Aldape K., Diamandis P. (2017). Precision histology: How deep learning is poised to revitalize histomorphology for personalized cancer care. NPJ Precis. Oncol..

[66] Men K., Chen X., Zhang Y., Zhang T., Dai J., Yi J., Li Y. (2017). Deep deconvolutional neural network for target segmentation of nasopharyngeal cancer in planning computed tomography images. Front. Oncol..

[67] Jethanandani A., Lin T.A., Volpe S., Elhalawani H., Mohamed A.S.R., Yang P., Fuller C.D. (2018). Exploring applications of radiomics in magnetic resonance imaging of head and neck cancer: A systematic review. Front. Oncol..

[68] Kerhet A., Small C., Quon H., Riauka T., Schrader L., Greiner R., Yee D., McEwan A. (2010). Application of machine learning methodology for PET-based definition of lung cancer. Curr. Oncol..

[69] Chilamkurthy S., Ghosh R., Tanamala S., Biviji M., Campeau N.G., Venugopal V.K., Mahajan V., Rao P., Warier P. (2018). Deep learning algorithms for detection of critical findings in head CT scans: A retrospective study. Lancet.

[70] Akkus Z., Galimzianova A., Hoogi A., Rubin D.L., Erickson B.J. (2017). Deep learning for brain MRI segmentation: State of the art and future directions. J. Digit. Imaging..

[71] Choi H., Ha S., Kang H., Lee H., Lee D.S. (2019). Deep learning only by normal brain PET identify unheralded brain anomalies. EBioMedicine.

[72] Ardila D., Kiraly A.P., Bharadwaj S., Choi B., Reicher J.J., Peng L., Tse D., Etemadi M., Ye W., Corrado G. (2019). End-to-end lung cancer screening with three-dimensional deep learning on low-dose chest computed tomography. Nat. Med..

[73] Lundervold A.S., Lundervold A. (2019). An overview of deep learning in medical imaging focusing on MRI. Zeitschrift Medizin. Phys..

[74] Shen L., Margolies L.R., Rothstein J.H., Fluder E., McBride R., Sieh W. (2019). Deep learning to improve breast cancer detection on screening mammography. Sci. Rep..

[75] Urban G., Tripathi P., Alkayali T., Mittal M., Jalali F., Karnes W., Baldi P. (2018). Deep learning localizes and identifies polyps in real time with 96% accuracy in screening colonoscopy. Gastroenterology.

[76] Kumar V., Gu Y., Basu S., Berglund A., Eschrich S.A., Schabath M.B., Forster K., Aerts H.J.W.L., Dekker A., Fenstermacher D. (2012). Radiomics: The process and the challenges. Magn. Reson. Imaging.

[77] Lambin P., Rios-Velazquez E., Leijenaar R., Carvalho S., van Stiphout R.G.P.M., Granton P., Zegers C.M.L., Gillies R., Boellard R., Dekker A. (2012). Radiomics: Extracting more information from medical images using advanced feature analysis. Eur. J. Cancer.

[78] Fan M., Li H., Wang S., Zheng B., Zhang J., Li L. (2017). Radiomic analysis reveals DCEMRI features for prediction of molecular subtypes of breast cancer. PLoS ONE.

[79] Skogen K., Schulz A., Dormagen J.B., Ganeshan B., Helseth E., Server A. (2016). Diagnostic performance of texture analysis on MRI in grading cerebral gliomas. Eur. J. Radiol.

[80] Hunter L.A., Chen Y.P., Zhang L., Matney J.E., Choi H., Kry S.F., Martel M.K., Stingo F., Liao Z., Gomez D. (2016). NSCLC tumor shrinkage prediction using quantitative image features. Comput. Med. Imaging Graph..

[81] Rao A., Rao G., Gutman D.A., Flanders A.E., Hwang S.N., Rubin D.L., Colen R.R., Zinn P.O., Jain R., Wintermark M. (2016). A combinatorial radiographic phenotype may stratify patient survival and be associated with invasion and proliferation characteristics in glioblastoma. J. Neurosurg..

[82] Mattonen S.A., Palma D.A., Haasbeek C.J., Senan S., Ward A.D. (2013). Distinguishing radiation fibrosis from tumour recurrence after stereotactic ablative radiotherapy (SABR) for lung cancer: A quantitative analysis of CT density changes. Acta Oncol..

[83] Scalco E., Fiorino C., Cattaneo G.M., Sanguineti G., Rizzo G. (2013). Texture analysis for the assessment of structural changes in parotid glands induced by radiotherapy. Radiother. Oncol..

[84] Basler L., Gabryś H.S., Hogan S.A., Pavic M., Bogowicz M., Vuong D., Tanadini-Lang S., Foerster R., Kudura K., Huellner M.W. (2020). Radiomics, tumor volume, and blood biomarkers for early prediction of pseudoprogression in patients with metastatic melanoma treated with immune checkpoint inhibition [published online ahead of print. Clin. Cancer Res..

[85] Shafiee M.J., Wong A. (2017). Discovery Radiomics via Deep Multi-Column Radiomic Sequencers for Skin Cancer Detection. J. Comput. Vis. Im. Syst..

[86] Szegedy C., Vanhouke V., Ioffe S., Shlens J., Wojna Z. (2015). Rethinking the Inception Architecture for Computer Vision. arXiv.

[87] Tian Y., Liu Z., Tang Z., Li M., Lou X., Dong E., Liu G., Wang Y., Wang Y., Bian X. (2019). Radiomics analysis of dti data to assess vision outcome after intravenous methylprednisolone therapy in neuromyelitis optic neuritis. JMRI.

[88] Guo J., Liu Z., Shen C., Li Z., Yan F., Tian J., Xian J. (2018). MR-based radiomics signature in differentiating ocular adnexal lymphoma from idiopathic orbital inflammation. Eur. Radiol..

[89] Deng J., El Naqa I., Xing L. (2018). Editorial: Machine learning with radiation oncology big data. Front. Oncol..

[90] Capobianco E., Dominietto M. (2020). From medical imaging to radiomics: Role of data science for advancing precision health. J. Pers. Med..

[91] Wu J., Cui Y., Sun X., Cao G., Li B., Ikeda D.M., Kurian A.W., Li R. (2017). unsupervised clustering of quantitative image phenotypes reveals breast cancer subtypes with distinct prognoses and molecular pathways. Clin. Cancer Res..

[92] Wu J., Tha K.K., Xing L., Li R. (2018). Radiomics and radiogenomics for precision radiotherapy. J. Radiat. Res..

[93] Azodi C.B., Tang J., Shiu S.H. (2020). Opening the black box: Interpretable machine learning for geneticists. Trends Genet..

[94] Hu L.S., Yoon H., Eschbacher J.M., Baxter L.C., Dueck A.C., Nespodzany A., Smith K.A., Nakaji P., Xu Y., Wang L. (2019). Accurate patient-specific machine learning models of glioblastoma invasion using transfer learning. AJNR Am. J. Neuroradiol..

[95] Parekh V.S., Jacobs M.A. (2019). Deep learning and radiomics in precision medicine. Expert Rev. Precis Med. Drug Dev..

[96] Dominietto M., Pica A., Safai S., Lomax A.J., Weber D.C., Capobianco E. (2020). Role Of Complex Networks For Integrating Medical Images And Radiomic Features Of Intracranial Ependymoma Patients In Response To Proton Radiotherapy. Front. Med..

[97] Hu L.S., Hawkins-Daarud A., Wang L., Li J., Swanson K.R. (2020). Imaging of intratumoral heterogeneity in high-grade glioma. Cancer Lett..

[98] Rathore S., Iftikhar M.A., Gurcan M.N., Mourelatos Z. (2019). Radiopathomics: Integration of radiographic and histologic characteristics for prognostication in glioblastoma. arXiv.

[99] Rathore S., Niazi T., Iftikhar M.A., Chaddad A. (2020). Glioma grading via analysis of digital pathology images using machine learning. Cancers.

[100] Tian J., Fan X., Xu R., Sun Y.S., Yang G. (2020). ASO Author Reflections: Radiopathomics Strategy of Combing Multi-scale Tumor Information on Pretreatment to Predict the Pathologic Response to Neoadjuvant Therapy. Ann. Surg. Oncol..

[101] Zhou C., Sun H., Chan H.-P., Chughtai A., Wei J., Hadjiiski L., Kazerooni E. Differentiating invasive and pre-invasive lung cancer by quantitative analysis of histopathologic images. Proceedings of the Medical Imaging 2018: Computer-Aided Diagnosis. International Society for Optics and Photonics.

[102] Kaczmarowski A., Iczkowski K.A., Hurrell S.L., McGarry S.D., Jacobsohn K., Hall W.A., Hohenwalter M., See W., LaViolette P.S. Predictive cytological topography (PiCT): A radiopathomics approach to mapping prostate cancer cellularity. Proceedings of the ISMRM 25th Annual Meeting & Exhibition.

[103] ClinicalTrials.gov NCT04271657, RadioPathomics Artificial Intelligence Model to Predict nCRT Response in Locally Advanced Rectal Cancer (RPAI-pCR). NCT04271657.

[104] Github Radiopathomics-TRG-nCRT-LARC Radiopathomics: A Framework of Fusing Multi-Scale Images Information to Enrich Description of Tumor Heterogeneity for LARC Patients Prior to nCRT. https://github.com/StandWisdom/Radiopathomics-TRG-nCRT-LARC.

[105] Chaddad A., Daniel P., Sabri S., Desrosiers C., Abdulkarim B. (2019). Integration of radiomic and multi-omic analyses predicts survival of newly diagnosed idh1 wild-type glioblastoma. Cancers.

